# Antimicrobial activity of probiotics against oral pathogens around orthodontic mini-implants: an in vitro study

**DOI:** 10.1590/2177-6709.26.5.e2119350.oar

**Published:** 2021-10-15

**Authors:** Ivana da Silva LEMOS, Fernanda F. de Albuquerque JASSÉ, Selly Sayuri SUZUKI, Cristiane de Melo ALENCAR, Denise Nami FUJII, Joissi Ferrari ZANIBONI, Hideo SUZUKI, Aguinaldo Silva GARCEZ SEGUNDO

**Affiliations:** 1 Faculdade São Leopoldo Mandic, Departamento de Ortodontia (Campinas/SP, Brazil).; 2 Universidade Federal do Pará, Departamento de Dentística Restauradora (Belém/PA, Brazil).; 3 Universidade Estadual Paulista, Faculdade de Odontologia de Araraquara, Departamento de Odontologia Restauradora (São Paulo/SP, Brazil).; 4 Faculdade São Leopoldo Mandic, Departamento de Microbiologia Bucal, (Campinas/SP, Brazil).

**Keywords:** Microbiology, Oral hygiene, Probiotics

## Abstract

**Objective:**

The aim of this *in vitro* study was to evaluate the antimicrobial effect of five types of non-industrialized and industrialized probiotics on biofilms formed around orthodontic mini-implants. The null hypothesis tested was: there is no difference in the antimicrobial effect between the five types of probiotics tested around orthodontic mini-implants.

**Methods:**

For the experiment, 120 mini-implants were immersed for seven days in *Staphylococcus aureus* solution for biofilm formation, and were subsequently plated in culture medium containing probiotics. The mini-implants were divided into six different groups, according to the probiotic used: G1)*Lactobacillus casei*; G2)*Lactobacillus brevis*; G3)*Lactobacillus rhamnosus*; G4) Lactobacillus from fermented milk Yakult®; G5) Lactobacillus from fermented milk Batavito® and G6) without use of probiotic, as negative control. Qualitative and quantitative analyses of all groups were performed using the CFU (colony forming unit) count.

**Results:**

The study showed that groups G4 and G6 did not present antimicrobial activity, in comparison to groups G1, G2, G3, and G5 (*p*< 0.05), which demonstrated antimicrobial activity.

**Conclusion:**

The non-commercial probiotic bacteria, *Lactobacillus casei* and *Lactobacillus rhamnosus*, as well as commercially available fermented milk Batavito® presented promising results in the reduction of colonization of mini-implants by *S. aureus*. Therefore, the null hypothesis was rejected.

## RESUMO

**Objetivo:**

O objetivo do presente estudo *in vitro* foi avaliar o efeito antimicrobiano de cinco tipos de probióticos não industrializados ou industrializados em biofilmes formados ao redor de mini-implantes ortodônticos. A hipótese nula testada foi: não há diferença no efeito antimicrobiano entre os cinco tipos de probióticos testados em torno dos mini-implantes ortodônticos.

**Métodos:**

Para o experimento, as cabeças de 120 mini-implantes foram imersas por sete dias em solução de *Staphylococcus aureus* para formação de biofilme e, posteriormente, semeadas em meio de cultura contendo probióticos. Os mini-implantes foram divididos em seis grupos diferentes, de acordo com o probiótico utilizado: G1)*Lactobacillus casei*; G2)*Lactobacillus brevis*; G3)*Lactobacillus rhamnosus*; G4) lactobacilos do leite fermentado Yakult®; G5) lactobacilos do leite fermentado Batavito®; e G6) sem uso de probiótico, como controle negativo. As análises qualitativas e quantitativas de todos os grupos foram realizadas usando a contagem de UFCs (unidades formadoras de colônia).

**Resultados:**

O estudo mostrou que os grupos G4 e G6 não apresentaram atividade antimicrobiana, em comparação aos grupos G1, G2, G3 e G5 (*p*< 0,05), os quais demonstraram atividade antimicrobiana.

**Conclusão:**

As bactérias probióticas não comerciais, *Lactobacillus casei* e *Lactobacillus rhamnosus*, assim como o leite fermentado comercializado, Batavito®, apresentaram resultados promissores na redução da colonização dos mini-implantes por *S. aureus*. Portanto, a hipótese nula foi rejeitada.

## INTRODUCTION

The main advantage of mini-implant (MI) is better control, direction and strength of orthodontic forces. However, for the successful use of MIs, it is important to evaluate the clinical and radiographic characteristics of the patient, as well as the age, sex, and place of insertion, in order to obtain primary and secondary stability.^1^ ^,^ ^2^

Since MIs are in close contact with adjacent hard and soft tissues, infections can occur. The infectious process can lead to early biological complications, during the osseointegration process (mucositis), or delayed, by the induction of a peri-implant disease (peri-implantitis).^3^ ^,^ ^4^ Previous studies showed that MI loss generally occurs in the first two months after the insertion. According to Freitas et al,^5^ peri-implant inflammation contributes for secondary stability loss of orthodontic mini-implants. Other studies have shown that home care and oral hygiene are considered important factors for mini-implant success. Chronic inflammation caused by plaque retention can lead to mobility and loss of the orthodontic mini-implant.^6^ ^-^ ^8^

Peri-implant disease is infectious in nature, since bacterial biofilm is one of the main etiological factors.^9^ ^,^ ^10^ According to the study performed by Persson and Renvert,^11^ peri-implantitis is a polymicrobial infection, and titanium dental implants provide an adequate environment for the development of a complex microbial biofilm. The authors identified that the total bacterial load in peri-implantitis for seven species (*Tannerella forsythia*, *Porphyromonas gingivalis*, *Treponema socranskii*, *Staphylococcus aureus*, *Staphylococcus anaerobius*, *Streptococcus intermedius* and *Streptococcus mitis*) was approximately four times higher than in healthy implants.^11^ Among these bacteria, especially Staphylococcus species present high affinity for titanium surfaces.^12^

*S. aureus* is one of the most common pathogens that involve implant infection.^4^ It is known for its ability to adhere to almost any titanium surface and is found more often in sites with peri-implantitis than in healthy implants.^11^ ^,^ ^13^ Canullo et al.^14^ stressed that clinicians should keep in mind that, in the initial stage of healing, this pathogen can influence the immune response and lead to peri-implant bone loss. 

In this sense, recent researches have suggested that the administration of probiotics may benefit oral health by preventing the growth of harmful microorganisms common to dental biofilm.^15^ ^,^ ^16^ Several studies^17^ ^-^ ^21^ have investigated the effects of oral use of probiotics on cariogenic microbiota. However, their effects on the prevention of periodontal disease and, more specifically, the biofilm around MIs is a subject to be explored. In this context, the aim of this study was to investigate the antimicrobial efficacy of five types of non-industrialized and industrialized probiotics on *S. aureus* biofilms formed around orthodontic MIs. The null hypothesis tested was: there is no difference in the antimicrobial effect between the five types of probiotics tested around orthodontic mini-implants.

## MATERIAL AND METHODS

### SAMPLE SIZE

A pilot study (n = 15) was performed to define the sample size. Considering a statistical power of 80%, α error of 5% and predicting a sample loss of 20% at the end of the study, the calculated sample size defined was twenty specimens per group. GPower^®^ software (Heinrich-Heine-Universität, Düsseldorf, Germany) was used to calculate the sample size, using the average values obtained in the pilot study.

### MINI-IMPLANTS

The sample comprised 120 mini-implants 12.0-mm long, with a diameter of 2.0 mm, 8.0-mm long screw thread, transmucosal length of 4 mm, 3.3-mm long head, obtained in dental supplies and used as received from Dat Steel (Comércio de Produtos Odontológicos Ltda, São Bernardo do Campo/SP, Brazil). The mini-implants are composed of surgical steel alloy, according to ASTMS-F138 standard (manufacturer’s specifications).

### BACTERIAL STRAINS AND CULTURE CONDITIONS

The probiotic strains used in this study were *Lactobacillus casei* (ATCC 393), *Lactobacillus brevis* (ATCC 367), and *Lactobacillus rhamnosus* (ATCC 9595) that were provided by Adolfo Lutz Institute (São Paulo, Brazil). The standard pathogenic bacterium *Staphylococcus aureus* (ATCC 25923) was used for the formation of biofilm. Strains were maintained at -80°C in 15% (w/w) glycerol. Lactic acid bacteria strains were grown in De Man, Rogosa and Sharpe (MRS) broth, while pathogenic strains were grown in tryptic soy broth (TSB, Oxoid). All strains were inoculated from stock culture and incubated for 24-48 hours at 37°C under microaerophilic conditions (5% CO_2_).

The industrialized probiotics tested were the fermented milk brands Yakult^®^ (Yakult S/A Indústria e Comércio, SP, Brazil), which contains a single probiotic bacterial species, *Lactobacillus casei Shirota;* and Batavito^®^ (BRF S.A., Carambeí, Brazil), which contains a combination of three probiotic bacteria (*Lactobacillus acidophilus, Bifidobacterium sp.,* and *Lactobacillus paracasei).* The products were purchased at a supermarket and stored under refrigeration as recommended by the manufacturers. For both brands, composition information of the probiotic strains, storage conditions, and shelf-life dates were provided by the manufacturers.

### STUDY DESIGN

The variable under study was the antimicrobial activity of five different probiotics against an oral pathogen (*Staphylococcus aureus*). The miniscrews were divided into six different experimental groups (n = 20) according to the probiotic used: G1)*Lactobacillus casei*; G2)*Lactobacillus brevis*; G3)*Lactobacillus rhamnosus*; G4) probiotics from the fermented milk Yakult^®^; and G5) probiotics from the fermented milk Batavito^®^. To verify the antimicrobial response to extremes, another group was added: G6) without the use of probiotic, as negative control.

### EXPERIMENTAL TRIAL

The mini-implants were pre-sterilized by gamma-cobalt 60 rays, with a minimum dose of 15 kGy and a maximum dose of 30 kGy, by the manufacturer. The mini-implants’ heads were fixed on a custom-made holder, to stabilization, and were immersed in a broth culture of *Staphylococcus aureus* for 7 days*.* Every 24 hours, the broth was removed to allow biofilm growth on the mini-implants surfaces. The mini-implants were washed with 5 ml sterile saline to eliminate planktonic bacteria, placed in sterile tubes (Eppendorf 1.5 ml) and vortexed for 30 seconds to remove the biofilm. The bacterial solution obtained was serially diluted, incubated in petri dishes with the brain heart infusion (BHI) medium for 24 hours in an incubator, and the number of colony forming units per ml (CFU/ml) in each group was counted.

### STATISTICAL ANALYSIS

Statistical analysis was performed by BioEstat Version 5.0 (*Instituto de Desenvolvimento Sustentável de Mamirauá, Belém, Brazil).* The results were compared using one-way analysis-of-variance (ANOVA) test followed by Tukey’s post test. For comparison between the industrialized probiotics (Yakult^®^ and Batavito^®^), the unpaired *t*-test was applied. Considering the homoscedasticity of the groups, a significance level of 5% was considered in the analyses for both tests.

## RESULTS

**Figure 1:**
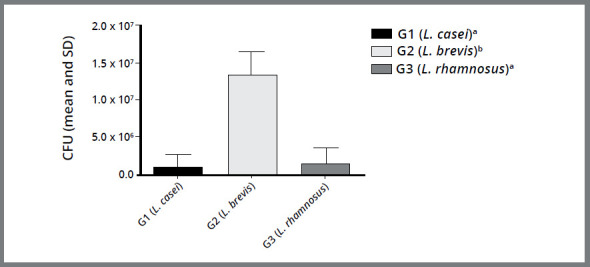
Column diagram of Colony Forming Unit values (mean and standard deviation) representing the antimicrobial activity of different probiotics (G1 -*Lactobacillus casei*, G2 -*Lactobacillus brevis* and G3 -*Lactobacillus rhamnosus*). Different superscript letters indicate statistically significant difference between groups (*p*< 0.05).

A comparison between the non-industrialized probiotics (G1, G2, and G3) is shown in Figure 1. It was observed that G1 and G3 possessed a more efficient antimicrobial activity than G2, with a statistically significant difference (*p*<0.05).

**Figure 2:**
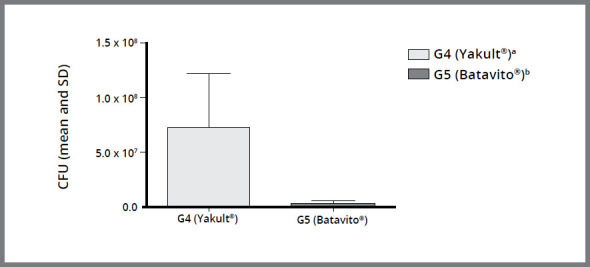
Column diagram of Colony Forming Unit values (mean and standard deviation) representing the antimicrobial activity of different industrialized probiotics (G4 - Lactobacillus from fermented milk Yakult^®^ and G5 - Lactobacillus from fermented milk Batavito^®^). Different superscript letters indicate statistically significant difference between groups (*p*< 0.05).

**Figure 3:**
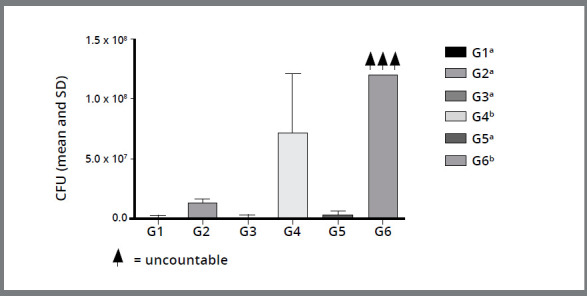
Column diagram of Colony Forming Unit values (mean and standard deviation) representing the antimicrobial activity of different probiotics and negative control. Different superscript letters indicate statistically significant difference between groups (*p*< 0.05).

A comparison between the industrialized probiotics (G4 and G5) is shown in Figure 2. It was observed that G5 showed a more efficient antimicrobial activity than G4, with a statistically significant difference (*p*< 0.05). It can be observed in Figure 3 that G4 and G6 (Yakult^®^ and Negative Control, respectively) did not show antimicrobial activity, in comparison to the other groups, with a statistically significant difference, i.e. between G4, G6 and G1, G2, G3, G5 (*p*< 0.05).

## DISCUSSION

The mechanism of action of probiotic therapy is by promoting the substitution of harmful pathogens present in a given microbiota by other non-pathogenic.^22^ ^,^ ^23^ According to Bosch et al,^15^ probiotic strains are able to perform better in an environment similar to their environment of origin. Based on this prerogative, *Lactobacillus casei* and *Lactobacillus brevis* were used in the present study, which can be isolated from human saliva. *Lactobacillus rhamnosus* was chosen because there are several studies in the literature evaluating their effect on cariogenic oral microbiota.^17^ ^-^ ^19^

In addition to isolated probiotic strains, this *in vitro* study evaluated the antimicrobial activity of two commercial brands of fermented milk containing probiotics, against the oral pathogen *Staphylococcus aureus.* Although these products were not developed for the purpose of controlling the oral pathogenic microbiota, Batavito^®^ fermented milk decreased the counts of the investigated microorganism, proving promising role in the prevention of peri-implantitis around mini-implants. These findings corroborate with the results of previous studies.^17^ ^,^ ^18^ ^,^ ^20^ ^,^ ^21^ Even though the mentioned studies showed a reduction of cariogenic microorganisms such as *S. mutans*, the present findings suggest a broader performance of probiotics against another oral pathogen, i.e. *Staphylococcus aureus.*

A possible explanation for the superior antimicrobial activity of Batavito was previously reported by Lodi et al.^24^ who attributed the difference in antimicrobial activity to its composition, since the fermented milk Batavito^®^ is composed of a mixture of three probiotic bacteria, while Yakult contains only a single bacteria i.e. *L. casei Shirota* ^®^. The simultaneous administration of different probiotics may affect the balance of oral ecosystem in an additive, cumulative, or competitive manner.^25^

In this study, when only non-commercial strains were analyzed, it was observed that *Lactobacillus casei* and *Lactobacillus rhamnosus* showed more efficient antimicrobial activity, compared to *Lactobacillus brevis*. The lower efficiency of *Lactobacillus brevis* can be explained by the findings of Bosch et al.,^15^ who observed the inability of this probiotic bacterium to form aggregates and co-aggregates with other microorganisms. It is important to emphasize that the aggregation activity could inhibit or reduce biofilm formation by pathogenic bacteria.^26^ In the same study, *Lactobacillus casei* showed the highest aggregation capacity, among the 48 species tested.^15^

The effect of surfactants obtained from three strains of *L. acidophilus* on adhesion and biofilm formation by *S. aureus* was analyzed by Walencka et al.^27^ They obtained positive results in terms of the inhibition caused by the surfactants tested. The inhibition probably occurs due to the influence of probiotic surfactants on the hydrophobicity of the surface of staphylococcal cells.^28^

Considering that some strains of *Lactobacillus* can induce caries, it is important to evaluate the cariogenic potential of each species of the probiotic bacteria tested in the present study.^17^ ^,^ ^22^ Although, Lodi et al.^24^ observed that fermented milk Batavito^®^ exhibited a protective effect against demineralization of dental enamel.

Despite promising results of the effect of probiotics on biofilm prevention around mini-implants, probiotic therapy should be used as an adjunct to oral hygiene techniques such as brushing and/or topical antimicrobial use. Despite the positive findings, animal model studies and controlled clinical trials should be performed to verify the *in vivo* effect of probiotics in patients undergoing orthodontic therapy with mini-implants.

## CONCLUSION

The findings of this study demonstrated that non-commercial probiotic bacteria, *Lactobacillus casei* and *Lactobacillus rhamnosus*, as well as commercially fermented milk Batavito^®^ presented promising results in the reduction of colonization of mini-implants by *S. aureus*. Therefore, the null hypothesis was rejected.
